# Direct discharge of patients with simple stable musculoskeletal injuries as an alternative to routine follow-up: a systematic review of the current literature

**DOI:** 10.1007/s00068-021-01784-z

**Published:** 2021-09-16

**Authors:** T. H. Geerdink, J. Verbist, J. M. van Dongen, R. Haverlag, R. N. van Veen, J. C. Goslings

**Affiliations:** 1 Department of Trauma Surgery, OLVG Hospital, Jan Tooropstraat 164, 1061 AE Amsterdam, The Netherlands; 2 Department of Health Sciences, Faculty of Science, Amsterdam Movement Sciences Research Institute, Vrije Universiteit, Amsterdam, The Netherlands

**Keywords:** Direct discharge, Fracture management, Systematic review, Healthcare utilization, Value-based healthcare, Fracture

## Abstract

**Purpose:**

There is growing evidence that patients with certain simple stable musculoskeletal injuries can be discharged directly from the Emergency Department (ED), without compromising patient outcome and experience. This study aims to review the literature on the effects of direct discharge (DD) of simple stable musculoskeletal injuries, regarding healthcare utilization, costs, patient outcome and experience.

**Methods:**

A systematic review was performed in Medline, Embase, CINAHL, Cochrane Library and Web of Science using PRISMA guidelines. Comparative and non-comparative studies on DD of simple stable musculoskeletal injuries from the ED in an adult/paediatric/mixed population were included if reporting ≥ 1 of: (1) logistic outcomes: DD rate (proportion of patients discharged directly); number of follow-up appointments; DD return rate; (2) costs; (3) patient outcomes/experiences: functional outcome; treatment satisfaction; adverse outcomes; other.

**Results:**

Twenty-six studies were included (92% conducted in the UK). Seven studies (27%) assessed functional outcome, nine (35%) treatment satisfaction, and ten (38%) adverse outcomes. A large proportion of studies defined DD eligibility criteria as injuries being minor/simple/stable, without further detail. ED DD rate was 26.7–59.5%. Mean number of follow-up appointments was 1.00–2.08 pre-DD, vs. 0.00–0.33 post-DD. Return rate was 0.0–19.4%. Costs per patient were reduced by €69–€210 (ranging from − 38.0 to − 96.6%) post-DD. Functional outcome and treatment satisfaction levels were ‘equal’ or ‘better’ (comparative studies), and ‘high’ (non-comparative studies), post-DD. Adverse outcomes were low and comparable.

**Conclusions:**

This systematic review supports the idea that DD of simple stable musculoskeletal injuries from the ED provides an opportunity to reduce healthcare utilization and costs without compromising patient outcomes/experiences. To improve comparability and facilitate implementation/external validation of DD, future studies should provide detailed DD eligibility criteria, and use a standard set of outcomes.

Systematic review registration number: 120779, date of first registration: 12/02/2019.

**Supplementary Information:**

The online version contains supplementary material available at 10.1007/s00068-021-01784-z.

## Introduction

### Rationale

Traditionally, all patients with musculoskeletal injuries are referred to a fracture clinic for further review and treatment, after initial assessment in an Emergency Department (ED). Consequently, fracture clinics are often characterized by the referral of large numbers of unselected patients, many of whom have minor injuries that do not require intervention. This leads to long waiting times, recurrent unnecessary reviews, and a high workload that inevitably has consequences for patient experience, staff morale, training, and quality of care [1].

A Virtual Fracture Clinic (VFC) model has been introduced in several hospitals worldwide as an alternative model of fracture care to regulate access to fracture clinics [2, 3]. This model is increasingly used in the United Kingdom (UK), but also in the Netherlands, Norway, Australia and New Zealand [3]. The VFC model comprises two main components [2].

First, direct discharge (DD) of patients with relatively simple stable musculoskeletal injuries. This means patients are discharged without subsequent review or repeated imaging, supported by self-removable orthoses, discharge leaflets, and a telephone helpline. Second, the establishment of an individualised management plan for all other patients during a daily consultant-led VFC review. This process should further streamline outpatient care and ensure that each patient is seen at the right time by the most appropriate person [2].

The DD protocols were developed based on studies showing that for several minor self-limiting injuries, casting offers no benefit over functional treatment [2], and on the assumption that patients with these injuries require reassurance and information, but do not need to attend a fracture clinic routinely. However, for DD to be a useful and acceptable alternative to routine follow-up, patient outcome, patient experience and complication rates should at least remain comparable, while healthcare utilizations and consequently costs are ideally reduced. Despite several independent studies that were conducted since DD was first established in 2011 [2], an overview of all current evidence regarding DD is not available.

### Objectives

The objective of this study was to systematically review the literature on the logistic and financial benefits of DD of patients with simple stable musculoskeletal injuries, as well as their patient-reported outcome, experience, and adverse outcomes. To illustrate the possible treatment of a patient with a simple stable musculoskeletal injury, both before and after DD, two case samples are provided below.

**Table:** 

Case example I—Torus/buckle fracture	
A 10-year-old boy presents to the Emergency Department (ED) complaining of wrist pain after a fall from his bicycle. The patient is examined by an ED physician or Orthopaedic consultant. Radiographic imaging of the wrist reveals a torus/buckle type fracture of the distal radius, without any angulation	
Treatment before implementation of direct discharge	Treatment after implementation of direct discharge
A plaster cast/splint is applied in the ED A follow-up appointment is scheduled in the fracture clinic in 7 days After 7 days, the cast/splint is removed. Bandage and a sling are applied. Parents are instructed to remove the bandage in a few days as pain allows. No further imaging is performed The patient is then discharged from follow-up with instructions regarding sports, etc	A removable wrist orthosis is applied in the ED Verbal instructions are provided in the ED with regard to the injury, recovery, when to remove the orthosis, when to contact the hospital, etc This is also summarized in a discharge leaflet and/or smartphone application No follow-up appointments are scheduled It is allowed to remove the orthosis e.g., to take a shower, and parents are instructed to permanently remove the orthosis after 7 days If pain does not allow, then the orthosis can be used for another week A special telephone helpline is available in case of any questions or concerns. If necessary, a face-to-face follow-up appointment is scheduled

**Table:** 

Case example II—Fifth metatarsal fracture	
A 50-year-old woman presents to the Emergency Department (ED) complaining of pain on the lateral side of her foot after missing the last step of the stairs. The patient is examined by an ED physician or Orthopaedic consultant. Radiographic imaging of the foot reveals a non-displaced fracture of the base of the fifth metatarsal bone (i.e., Zone 1/Dancer’s fracture)	
Treatment before implementation of direct discharge	Treatment after implementation of direct discharge
A plaster cast/splint is applied in the ED A follow-up appointment is scheduled in the fracture clinic in 7 days After 7 days, the cast/splint is removed and a new splint is applied. The patient is scheduled for another appointment in 5 weeks After 5 weeks, the splint is removed and radiographic imaging is performed. The radiograph shows first signs of bone healing Based on local protocols and physician preference/examination, the patient is then either; discharged from further follow-up with instructions regarding sports, etc reviewed again in a few weeks to assess functional outcome and perform radiographic imaging	A removable orthosis (walker boot) is applied in the ED Verbal instructions are provided in the ED with regard to the injury, recovery, when to remove the walker, when to contact the hospital etc This is also summarized in a discharge leaflet and/or smartphone application No follow-up appointments are scheduled It is allowed to remove the orthosis e.g., to take a shower, patients are instructed to use the walker for 6 weeks and wear a supportive shoe A special telephone helpline is available in case of any questions or concerns. If necessary, a face-to-face follow-up appointment is scheduled, and/or imaging is performed

## Methods

### Protocol and registration

This systematic review was planned, conducted, and reported using PRISMA guidelines [4]. A study protocol was registered with the PROSPERO register prior its commencement (registration number: 120779) [5].

### Eligibility criteria

Both comparative (i.e., routine care before DD protocols were adopted compared to DD) and non-comparative studies (i.e., a DD cohort only) were considered if they featured DD of one or more musculoskeletal injuries in an adult, paediatric, or mixed population. Case reports and abstracts were excluded. There were no restrictions regarding the timing of the study, nor the duration of follow-up. Only articles reported in English were included.

**Fig. 1:**
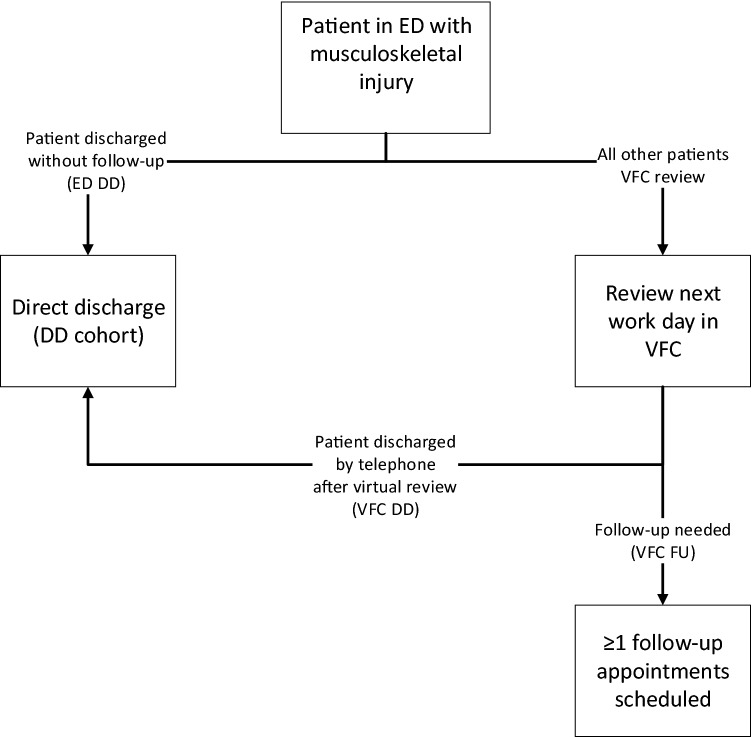
Virtual Fracture Clinic model, explaining the difference between patients discharged directly from the Emergency Department (ED DD), or after virtual review (VFC DD). *ED* emergency department, *VFC* virtual fracture clinic

Direct discharge was defined as scheduling no routine follow-up appointment after the ED visit. This could either take place directly after the ED visit (ED DD) or after a daily ‘virtual’ review (VFC DD, Fig. 1). A single fracture clinic visit, shortly after attending the ED, was interpreted as ED DD if the sole purpose of this visit was application of a removable splint/orthosis. Studies were excluded if any further information or assessments were part of this visit. Studies were also excluded if reporting the potential effects of DD, without actually discharging patients directly. Both prospective and retrospective studies were included if reporting one of the following outcomes:

### Logistic outcomes

Logistic outcomes included: (1) proportion of patients discharged directly, (2) number of follow-up appointments, and (3) number of repeat radiographs during follow-up. If the number of follow-up appointments was not reported, a study was also included if it reported a return rate instead (i.e., the proportion of patients that returned to the hospital despite being discharged directly).

### Financial outcomes

Financial outcomes included any report on costs, for instance healthcare costs, societal costs etc.

### Patient outcomes

Patient outcomes included any patient-reported experience/outcome measures (PREMs/PROMs) and adverse outcomes.

### Information sources

Medline, Embase, CINAHL, Cochrane Library and Web of Science databases were searched from inception to 15 January 2020. A limited update literature search was performed on 6 August 2020. Reference lists of included studies were scanned to ensure literature saturation.

### Search

The electronic search strategy was developed by a health librarian and peer-reviewed by another librarian. Medical subject headings (MeSH) were used in Medline and complemented by text words related to DD and (virtual) fracture clinic redesign. This search strategy was then translated for the other databases. The PICO strategy and the full electronic search of Medline are reported in Tables S1 and Table S2, respectively.

### Study selection

Two review authors (THG and JV) independently screened the titles and abstracts yielded by the search against the inclusion criteria. Full texts were obtained if studies appeared to meet the inclusion criteria, or in case of uncertainty. All reasons for exclusion were recorded. Reviewing authors were not blinded to the journal titles, study authors and institutions.

### Data collection process

A data extraction sheet was developed and pilot-tested on five randomly selected included studies, and refined accordingly. Data from the included studies were extracted by one author (THG) and checked by another (JV).

### Data items

The following data items were collected for all included studies: country; year; design; sample size; injury/injuries studied; eligibility criteria for DD; population (adult/paediatric); study period. If reported, the distribution of patients across the various parts of the VFC model (i.e., ED DD; VFC DD or VFC follow-up, Fig. 1) was extracted and summarized in Table S3. Information on the type of immobilization used before and after DD protocols were implemented was extracted and summarized in Table S4.

The following data items were extracted if available: the proportion of patients discharged directly; number of follow-up appointments; return rate; number of repeat radiographs; costs; functional outcome score; any other PROMs or PREMs measured; any adverse outcomes reported. Method of assessment, timing and response rate (if applicable) were collected for all outcomes of interest. Missing information was scored as ‘not reported’. Authors were contacted if further information or confirmation of data was required.

In some studies, only part of the intervention cohort consisted of patients who were discharged directly (e.g., if the effects of the whole VFC model as a whole was assessed, rather than the effect of DD in particular; Fig. 1). For those studies, if possible, we extracted logistic outcome and adverse outcome data only for the patients who were discharged directly (DD cohort). If this was not possible, data were only extracted if > 75% of patients within the intervention cohort were discharged directly. Costs and PREMs/PROMs data were extracted and only included in the main analysis if reported specifically for patients discharged directly (DD cohort).

### Risk of bias in individual studies

Two reviewers (THG and JV) independently assessed risk of bias using the Cochrane risk-of-bias tool for randomized controlled trials [6], and the Newcastle–Ottawa Quality Assessment Scale (NOS) for non-randomized studies [7]. A modified NOS was used to assess non-comparative studies. Following the manuals of the tools, studies were scored as either having a “low”, “medium”, “high” or “unclear” risk of bias.

Disagreements regarding study selection, data collection or risk-of-bias assessment were resolved through discussion or by consulting a third author (JCG).

### Summary measures

To summarize the results, various outcome-specific summary measures were estimated based on the extracted data, including: (1) logistic outcomes: DD rate (proportion of patients discharged directly); mean number of follow-up appointments, and in case of a comparative study the mean reduction; mean repeat radiographs, (2) financial outcomes: costs as reported, and in case of a comparative study, the absolute and proportion difference in euro (€) and %, respectively, (3) patient outcomes: functional outcome score using a validated multi-item questionnaire; satisfaction with treatment; number and rate of adverse outcomes divided in non-union, secondary surgery, and other.

### Synthesis of results

We chose not to pool data via meta-analysis due to high levels of clinical and methodological heterogeneity. For all outcomes, findings were, therefore, presented narratively and in summary tables.

## Results

### Study selection

**Fig. 2:**
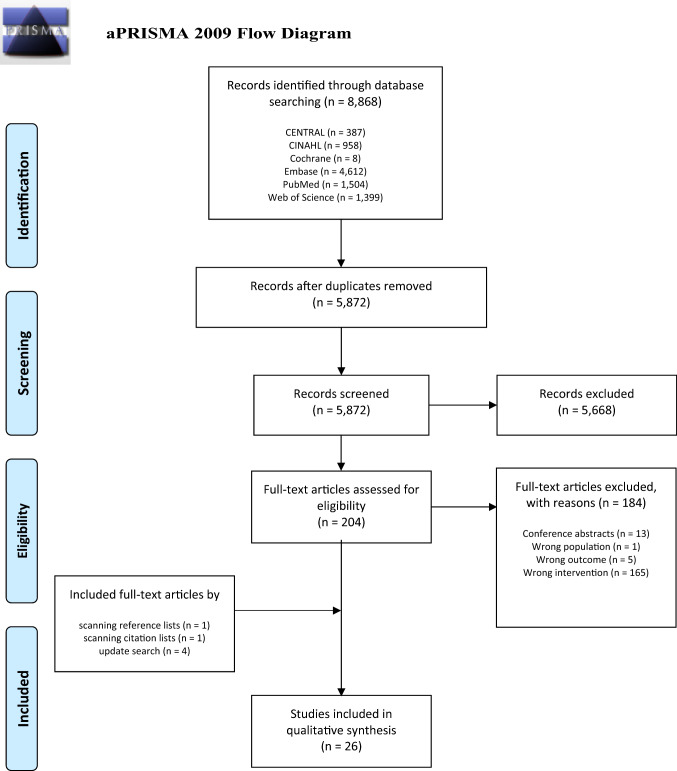
Flowchart depicting article screening and inclusion

The search identified 5872 unique records, 5668 of which were excluded after screening title and abstract. A further 184 studies were excluded after reading full texts. Six additional studies were included after scanning reference/citation lists and an updated search. Figure 2 shows the selection process and an overview of the reasons for exclusion.

Two studies were based on the same cohort [2, 8]. In both studies, the only outcome relevant for this systematic review was the DD rate. Therefore, we did not exclude one as duplicate, but merged the results of these studies in the corresponding table. Another study described results for three types of injuries separately [9]. Therefore, the results of this study were summarized per injury separately in all tables.

### Study characteristics

Ten studies (38%) compared DD to routine care [8–17], whereas 16 (62%) studies did not have a control cohort [1, 2, 18–31]. Twenty-four studies (92%) were conducted in the UK and two in New Zealand (8%) [25, 26]. The patient population was described in 22 studies (85%), whereas 4 (15%) did not describe the study population. One study included an adult population, seven a paediatric population, and 14 a mixed population. Twelve studies included a single injury (46%) and 14 (54%) multiple injuries. The 26 studies included a total of 38,506 patients, 3832 of which were assessed before implementing any changes (control cohort) and 34,674 patients thereafter (intervention cohort). Of these 34,675 patients, 11,133 were discharged directly (DD cohort).

A detailed description of the eligibility criteria for DD was reported in ten studies (Table 1) [9–11, 13, 16, 17, 25, 26, 28, 31].

**Table 1:** Baseline characteristics of included studies

Study details				Sample size; *n*		Study population			Outcomes						
First author	Country	Year	Design	Control	Intervention	Injuries studied	Described eligibility criteria for DD	Population	D	L	€	F	S	AO	Period; m
Comparative															
Abdelmalek [10]	England	2015	RCSPPP	36	27	5MC neck Fx	Angulation < 50°, no rotational deformity	A + P	+	+	−	−	−	−	6 vs. 6
Bansal [11]	England	2007	PCSPPP	40	38	5MC neck Fx	Angulation < 70°, no rotational deformity, no delay (< 1w)	≥ 16y	−	+	−	+	+	−	NR
Ferguson [12]	Scotland	2015	RPPRCS	279	339	5MT Fx	Not clearly defined	A + P	+	+	−	−	−	+	12 vs. 12
Hamilton [13]	England	2013	RCT	158	159	Stable forearm Fx	Torus/greenstick: < 15° angulation; SH I/II: < 5 mm displaced	P ≥ 2y	−	+	+	+	+	−	34
Kelly [14]	Ireland	2019	RPPRCS	NR	247	Multiple	Not clearly defined	A + P	+	−	−	−	−	−	6 vs. 6
Khan [15]	Ireland	2007	RCT	48	69	Torus Fx distal radius	Not clearly defined, described as ‘stable’	P	−	−	−	−	+	−	3
Mackenzie [9]	Scotland	2018	RPPCS	108	88	5MC Fx	Isolated extra-articular Fx	A + P ≥ 13y	−	+	+	+	+	+	6 vs. 6
				111	87	5MT Fx	Any	A + P ≥ 13y	−	+	+	+	+	+	6 vs. 6
				118	114	Radial head/neck Fx	Mason type 1 and 2	A + P ≥ 13y	−	+	+	+	+	+	6 vs. 6
Matthews [16]	England	2014	RPPCS	55	23	Clavicle Fx	Isolated single fragment midshaft Fx	P 1-15y	+	−	−	−	+	−	10 vs. 3
Seewoonarain[17]	England	2019	RPPCS	39	44	Torus Fx distal radius	No associated ulnar styloid Fx	P < 16y	+	+	+	−	−	−	6. vs 6
Vardy [8]	Scotland	2014	RPPCS	2840	6385	All not requiring immediate inpatient treatment	Not clearly defined, included 5MC neck, paediatric greenstick/torus, clavicle, mallet finger, radial head, 5MT Fx	A + P	+	−	−	−	−	−	12 vs. 12
Non-comparative															
Bhattacharyya[18]	Scotland	2017	RCS		138	Clavicle Fx	Not clearly defined, mostly Robinson 2a1 and 3a1	A + P	+	+	−	+	−	+	12
Breathnach [19]	Ireland	2018	RCS		157	‘Orthopaedic Fx’	Not clearly defined, includes buckle Fx/STI of hand, foot, ankle	A + P	+	−	−	−	−	+	1.5
Brogan [20]	England	2017	CS		663	Suspected 5MT Fx	All 5MT Fx, but ‘Jones’ Fx’ based on clinician’s decision	A + P	+	+	−	−	−	+	24
Brooksbank[21]	Scotland	2014	PCS		47	Mallet finger	Not clearly defined	NR	+	+	−	+	+	−	12
Callender [22]	Ireland	2015	RCS		119	Wrist torus Fx	Not clearly defined	P	−	+	+	−	−	+	12
Evans [23]	England	2018	PCS		291	Hand and wrist injuries	‘Simple tuft, 5MC neck, minor avulsion, volar plate and STI’	A + P	+	−	−	−	−	+	4.5
Gamble [24]	Scotland	2015	RCS		167	5MC Fx	Not clearly defined	A	−	+	−	+	+	−	6
Gleeson [25]—I	NZ	2016	RCS		61	Torus Fx radius/ulna; clavicle Fx	Torus: < 15° angulation; Clavicle: isolated Fx with bone contact	P > 1y	−	+	+	-	+	−	12
Gleeson [26]—II	NZ	2016	RCS		33	5MC neck Fx; 5MT base Fx; Ankle Fx, Weber type A	5MC: < 60° angulation, no rotation; 5MT: Zone 1 < 2 mm displaced, < 10% angulation; Weber A: < 4 mm displaced, fragment < 5 mm	NR	−	−	+	−	−	−	9
Ibrahim [27]	Scotland	2018	CS		100	Acute closed STI/bony hand injuries	Not clearly defined, but excl. isolated carpal and wrist Fx	NR	+	−	−	−	−	+	1.5
Jayaram [28]	Scotland	2014	RCS		182	Radial head/neck Fx	Unilateral, Mason type 1 or 2 or fatpad sign, no other injuries	NR	+	+	−	−	−	+	12
Jenkins [2]	Scotland	2014	RCS		6385	5MT, 5MC, distal radius, torus, radial head Fx, mallet finger, child clavicle Fx	Not clearly defined, described as ‘simple, self-limiting stable Fx’	A + P	+	−	−	−	−	−	12
Little [29]	England	2020	PCS		3709	Hand/wrist injuries	Stable STI/bony injury, no significant swelling or ROM problems	A + P	+	−	−	+	+	−	21
O’Reilly [30]	Ireland	2019	CS		2704	Simple 5MC, 5MT, clavicle, radial head or torus Fx, mallet finger	Not clearly defined: ‘simple injuries’, mallet finger: no bony injury	A + P	+	−	−	−	−	−	19
Robinson [31]	England	2017	PCS		229	MC, phalanx hand, distal radius/ulna, elbow, clavicle, metatarsal, lateral malleolus Fx	Metacarpal: < 60° angulation, no rotational deformity; phalanx: minor avulsion, wrist: undisplaced or torus, elbow: undisplaced/ fatpad (> 5y); clavicle: un/minor displaced, metatarsal: undisplaced; lateral malleolus: undisplaced (> 5y)	P	-	+	−	−	−	+	6
White [1]	Scotland	2017	RCS		12,069	All patients with musculoskeletal injuries	Mallet finger with no bony injury, 5MC neck, radial head/neck Mason type I or II, 5MT base Fx or Fx of lesser toe	A + P	+	−	−	−	−	−	12

Studies were included if they reported at least one of: D (proportion of patients discharged directly), L (logistic outcomes), € (financial outcomes), F (functional outcomes), S, (satisfaction with treatment); AO (Adverse outcomes)

*5MC* fifth metacarpal, *5MT* fifth metatarsal, *A* adult, *CS* cohort study, not mentioned specifically if data were collected retrospectively or prospectively. *DD* direct discharge, *Fx* fracture, *PCS* prospective cohort study, *PPP* prospective pre-post study, *m* months, *MC* metacarpal, *mm* millimetre, *NR* not reported, *NZ* New Zealand, *P* paediatric, *RCS* retrospective cohort study; *ROM* range of motion, *RPP* retrospective pre-post study, *SH* Salter-Harris, *STI* soft-tissue injury

### Risk of bias within studies

Risk of bias was “low” in fifteen studies, “high” in four studies and “unclear” in the remaining seven studies (Table S5).

## Results of individual studies

### Logistic outcomes

In 10 studies, the intervention cohort consisted exclusively of patients discharged directly. Hence, a DD rate could not be estimated. In the remaining 16 studies, the DD rate ranged from 18.2 to 97.9% (Table 2). Of these 16 studies, three reported a DD rate as a proportion of all patients that attended the ED with a musculoskeletal injury. The DD rate in these studies was 26.7%, 59.5% and 33.3%, respectively [1, 8, 30].

**Table 2:** Number of patients discharged directly in the intervention cohort

Study	Injury	DD cohort size; n	DD rate; %	Relative to all patients with
Comparative				
Abdelmalek [10]	5MC neck Fx	6	22.2	5MC neck fractures
Bansal [11]	5MC neck Fx	38	n/a	–
Ferguson [12]	5MT Fx	280	82.6	5MT fractures
Hamilton [13]	Paediatric forearm Fx	159	n/a	–
Kelly [14]	Multiple	45	18.2	Minor trauma injuries
Khan [15]	Torus Fx wrist	69	n/a	–
Mackenzie [9]	5MC Fx	88	n/a	–
	5MT Fx	87	n/a	–
	Radial head/neck Fx	114	n/a	–
Matthews [16]	Paediatric clavicle Fx	18	78.3	Paediatric zone 2 (midshaft) clavicle fractures
Seewoonarain [17]	Torus Fx wrist	33	75.0	Torus fractures of the distal radius
Vardy/Jenkins [2, 8]	Multiple	3802	59.5	Musculoskeletal injuries in the ED not requiring immediate admission
Non-comparative				
Bhattacharyya [18]	Clavicle Fx	62	44.9	Clavicle fractures
Breathnach [19]	Multiple	42	26.8	Any type of fracture
Brogan [20]	5MT Fx	499	75.3	5MT fractures
Brooksbank [21]	Mallet finger	46	97.9	Mallet finger injuries
Callender [22]	Torus Fx wrist	119	n/a	–
Evans [23]	Hand/wrist injuries	54	18.6	Hand/wrist injuries
Gamble [24]	5MC Fx	167	n/a	–
Gleeson [25]—I	Torus Fx wrist; paediatric clavicle Fx	61	n/a	–
Gleeson [26]—II	5MC neck, 5MT base, Weber A Fx	33	n/a	–
Ibrahim [27]	Hand/wrist injuries	38	38.0	Hand/wrist injuries
Jayaram [28]	Radial head/neck Fx	182	90.1	Mason type 1 or 2 radial head/neck fractures
Little [29]	Hand/wrist injuries	968	26.1	Hand injuries
O’Reilly [30]	Multiple	901	33.3	Musculoskeletal injuries in the ED
Robinson [31]	Multiple paediatric	229	n/a	–
White [1]	Multiple	3222	26.7	Musculoskeletal injuries in the ED

If studies reported exclusively on outcomes in patients that were discharged directly, a direct discharge rate could not be determined (n/a/)

*5MC* fifth metacarpal, *5MT* fifth metatarsal, *DD* direct discharge, *ED* emergency department, *FU* follow-up, *Fx* fracture, *n/a* not applicable, *VFC* virtual fracture clinic

The mean number of follow-up appointments was reported in eleven studies, and ranged from 1.00 to 2.08 in the control cohort vs. 0.0–0.33 after implementing DD (Table 3). In the comparative studies, the mean reduction of follow-up appointments ranged from 1.00 to 1.78 appointments per patient.

**Table 3:** Logistic outcomes

Study	Injury	Number of appointments; mean		Mean reduction	Returned after DD	Repeat radiographic images; mean	
		Control	DD		Rate; %	Control	DD
Comparative							
Abdelmalek [10]	5MC neck Fx	1.33	0.0	1.33	0.0	NR	0.0
Bansal [11]	5MC neck Fx	1.83	0.05	1.78	5.3	NR	NR
Ferguson [12]	5MT Fx	1.76	< 0.30*	1.46	2.5	NR	NR
Hamilton [13]	Paediatric forearm Fx	1.05	0.02	1.03	1.3	NR	NR
Mackenzie [9]	5MC Fx	1.08	0.08	1.00	NR	0.4	0.06
	5MT Fx	2.08	0.33	1.75	NR	0.3	0.13
	Radial head/neck Fx	1.25	0.22	1.03	NR	1.1	0.31
Seewoonarain [17]	Torus Fx wrist	1.00	0.0	1.00	0.0	1.0	0.0
Non-comparative							
Bhattacharyya [18]	Clavicle Fx		0.02		1.6		NR
Brogan [20]	5MT Fx		< 0.17**		NR		NR
Brooksbank [21]	Mallet finger		NR		19.4		NR
Callender [22]	Torus Fx wrist		0.0		0.0		0.0
Gamble [24]	5MC Fx		0.0		0.0		0.0
Gleeson [25]—I	Torus Fx wrist; clavicle Fx		0.0		0.0		0.0
Jayaram [28]	Radial head/neck Fx		NR		1.1		NR
Robinson [31]	Multiple		NR		9.2		NR

*5MC* fifth metacarpal, *5MT* fifth metatarsal, *DD* direct discharge, *Fx* fracture, *NR* not reported

*Was determined in a cohort of 339 patients, 82.6% of which were discharged directly

**Was determined in a cohort of 663 patients, 75.3% of which were discharged directly

Twelve studies determined a return rate of patients after DD. Brooksbank et al. found 19.7% of patients that sustained a mallet finger injury to return after DD [21]. All other studies reported a return rate of less than 10%.

The mean number of repeat radiographs was reported in five studies [9, 10, 17, 22, 24, 25]. Of the two comparative studies, Mackenzie et al. reported a mean reduction of 0.34, 0.17 and 0.79 radiographs per patient, in patients with fifth metacarpal neck, fifth metatarsal and radial head fractures, respectively [9]. Seewoonarain et al. found a mean reduction of 1.00 radiograph per patient with a torus wrist fracture [17]. In three non-comparative studies, the mean number of repeat radiographs in the DD cohort was 0.0 based on a return rate of 0% [22, 24, 25].

### Financial outcomes

Six studies estimated costs with reductions ranging from €69 to €210 per patient after implementing DD (Table 4) [9, 13, 17, 22, 25, 26].

**Table 4:** Financial outcomes in three comparative and three non-comparative studies

Study	Injury	How were costs calculated	Costs per patient before DD	Costs per patient after DD	Δ/patient; €	Δ/patient; %	Sig.
Comparative							
Hamilton [13]	Paediatric forearm Fx	Costs resulting from clinic visits, MIU visits, GP visits, telephone contacts, plaster room contact, use of immobilization materials	Mean GBP 261.04	Mean GBP 160.48	− €11,157	− 38.5%	< 0.001
Mackenzie [9]	5MC Fx	National Health Service secondary care cost analysis, including staffing, operation and radiology costs	Median GBP 139.83 (86.1–288.69)	Median GBP 12.17 (0.58–21.02)	− €14,164	− 91.3%	NR
	5MT Fx	Similar	Median GBP 297.74 (210.39–385.15)	Median GBP 113.35 (4.48–316.72)	− €204,58	− 61.9%	NR
	Radial head/neck Fx	Similar	Median GBP 167.11 (127.16–257.23)	Median GBP 28.97 (9.82–44.23)	− €15326	− 82.7%	NR
Seewoonarain [17]	Torus Fx wrist	Costs resulting from clinic visits and material costs, staffing costs	Mean GBP 163.82	Mean GBP 101.60	− €6903	− 38.0%	NR
Non-comparative							
Gleeson [25]—I	Torus Fx wrist; Paediatric clavicle Fx	Financial savings as quantified by business analyst. Unclear description of exact calculation		NZD − 379.88 per clavicle visit NZD − 223.88 per buckle visit	− €21,028 − €12,993	–^a^ –^a^	
Gleeson [26]—II	Multiple	Financial savings as quantified by business analyst. Unclear description of exact calculation		NZD -379.88 per visit	− €21,028	–^a^	
Callender [22]	Torus Fx wrist	Simple estimation of costs from outpatient clinic visits and soft cast material costs, compared to estimation of previous situation	Mean GBP 162.11	Mean GBP 5.50	− €17,376	− 96.6%	

Studies that reported on costs within the DD cohort. The difference between the cohort before implementing DD and after implementing DD were calculated per patient. The conversion rates to EUR (€) at the time of calculation were: 1 GBP = EUR 1.10949 and 1 NZB = EUR 0.553554. *5MC* fifth metacarpal, *5MT* fifth metatarsal, *DD* direct discharge, *EUR* Euro’s, *Fx* fracture, *GBP* Great Britain Pounds, *GP* general practitioner, *MIU* minor injury unit, *NR* not reported, *NZD* New Zealand Dollars, *RH* radial head, *Sig.* significance level

^a^Not reported as percentage as only post-DD costs, and no pre-DD costs were provided

### Patient outcomes

Seven studies assessed functional outcome using a validated questionnaire, including four non-comparative and three comparative studies (Table 5). The three comparative studies found equal functional outcome scores before and after implementing DD [9, 11, 13]. Of them, Mackenzie et al. reported significantly better QuickDASH scores at 6 months within the DD subgroup of patients with fifth metacarpal fractures [9]. All four non-comparative studies reported good recovery in terms of functional outcome based on QuickDASH scores within the DD cohort.

**Table 5:** Functional outcome assessed using a validated multi-item questionnaire

Questionnaire	Study	Injury	Assessment			Control		DD		Sig.
			Reported as	How	When	Score	Resp. rate; %	Score	Resp. rate; %	
Comparative										
DASH	Bansal [11]	5MC neck Fx	Mean score	Phone	12 w	8.1 (SD 6.0)	NR	6.8 (SD 4.9)	NR	NS
FADI	Mackenzie [9]	5MT Fx	Median score	NR	6 m	100 (95–100)	66.0	100 (97–100)	80.0	NS
CHAQ	Hamilton [13]	Paediatric forearm Fx	Mean change index score	Post	4 w	− 0.48 (SD 4.87)	67.1	0.48 (SD 4.02)	79.2	NS
QuickDASH	Mackenzie [9]	Radial head/neck Fx	Median score	NR	6 m	0.0 (0–2.3)	80.0	0.0 (0–4.5)	80.0	NS
	Mackenzie [9]	5MC Fx	Median score	NR	6 m	0.0 (0–7.9)	21.0	0.0 (0–0)	53.0	0.001
Non-comparative										
QuickDASH	Gamble [24]	5MC Fx	Median score	Post/phone	> 1 y			2.3 (0 to 6.8)	80.6	
	Brooksbank [21]	Mallet finger	Median score	Post	1 y			2.27 (0 to 4.55)	77.0	
	Bhattacharyya [18]	Clavicle Fx	Mean score	Post	1 y			16.1 (SD 25.2)	71.0	
QuickDASH modules Disability; Work; Sport	Little [29]	Hand/wrist injuries	Median module score	Post/phone	> 6 m			D: 4.4 (0–24) W: 14 (0–32) S: 14 (0–32)	92.0 89.0 38.0	

Studies that reported on any type of patient-reported functional outcome measure. *5MC* fifth metacarpal, *5MT* fifth metatarsal, *A* agreed, *CHAQ* Childhood Health Assessment Questionnaire, *DASH* disabilities of the arm, hand and shoulder, *DD* direct discharge, *FADI* Foot & Ankle Disability Index, *Fx* fracture, *m* months, *NR* not reported, *NS* not significant, *P* point, *RH* radial head, *S* satisfied, *SA* strongly agreed, *SD* standard deviation, *Sig.* significance level, *VS* very satisfied, *w* weeks, *y* year(s)

Satisfaction with treatment was reported by nine studies, five of which were comparative and four were non-comparative (Table 6). Of the comparative studies, Bansal et al. found that patients with fifth metacarpal neck fractures were more satisfied after DD compared to patients that were followed-up [5.1 vs. 7.0 on a 1 (very dissatisfied) to 10 (very satisfied) rating scale] [11]. Three studies reported no difference in satisfaction before and after implementing DD, without providing any rates [13, 15, 16]. Mackenzie et al. found high satisfaction rates before and after implementing DD (95% vs. 98% using a yes/no “are you satisfied with treatment” question) [9]. The four non-comparative studies all assessed satisfaction rates based on a Likert satisfaction scale, and satisfaction ranged from 84.9 to 100% [21, 24, 25, 29].

**Table 6:** Satisfaction with treatment before and/or after direct discharge

Experience measure	Study	Injury	Assessment			Control		DD		Sig.
			Assessed by	How	When	Score	Resp. rate; %	Score	Resp. rate; %	
Comparative										
Satisfaction with treatment	Bansal [11]	5MC neck Fx	Scale 1 (VD) to -10 (VS)	Phone	12 w	5.1 (SD 1.9)	NR	7.0 (SD 1.4)	NR	0.04
	Hamilton [13]	Paediatric forearm Fx	Seven domain satisfaction, Likert	Post	6 m	‘No difference’	93.7	‘No difference’	88.1	NS
	Matthews [16]	Clavicle Fx	NR	NR	> 6 m	NR	–	Majority satisfied	100	NR
	Khan [15]	Torus Fx wrist	Scale 1 (VD) to -10 (VS)	Phone	4–5 w	Highly satisfied	100	‘Highly satisfied’	100	NR
	Mackenzie [9]	5MC, 5MT, RHFx	Yes/no question	NR	6 m	95% S	56.4	98% S	72.3	NR
Non-comparative										
Satisfaction with treatment	Gamble [24]	5MC Fx	Likert scale	Post/phone	> 1 y			84.9% VS or S	59.0	
	Gleeson [25]—I	Torus Fx, clavicle Fx	Likert scale	Phone	3–6 w			100% VS or S	45.9	
	Brooksbank [21]	Mallet finger	Likert scale	Post	1 y			100% VS or S	77.0	
	Little [29]	Hand/wrist injuries	Likert scale	Post/phone	> 6 m			99.3% VS or S	94.0	

Studies that reported on satisfaction with treatment or treatment outcome. *5MC* fifth metacarpal, *5MT* fifth metatarsal, *Fx* fracture, *m* months, *NR* not reported, *NS* not significant, *S* satisfied, *SD* standard deviation, *Sig.* significance level, *VD* very dissatisfied, *VS* very satisfied, *w* weeks, *y* year(s)

All other PROMs and PREMs that were reported in the individual studies are summarized in Table S6. These included, amongst others, satisfaction with recovery and whether patients had visited other clinicians such as their general practitioner for the treatment of their injury.

Non-union-rate was reported by three studies (Table 7), ranging from 0.0 to 0.9% in the control cohort vs. 0.0–2.3% in the DD cohort [9, 12, 20]. Secondary surgery rates were reported in two comparative studies and three non-comparative studies, ranging from 0.0 to 1.1% in the control cohort [9, 12], [9, 12, 18, 20, 28] vs. 0.0–2.3% in the DD cohort [9, 12, 18, 20, 28]. Four non-comparative studies reported that ‘no adverse outcomes’ occurred [22, 23, 27, 31].

**Table 7:** Adverse outcomes

Outcome	Study	Injury	Assessment		Outcome; *n* (%), reason if available		Sig.
			How	When	Control	DD	
Comparative							
Non-union rate	Ferguson [12]	5MT Fx	EPR evaluation	> 1 y	1 (0.4), zone 1 fracture*	2 (0.6), one Jones, one proximal diaphyseal fracture*	NS
	Mackenzie [9]	5MC Fx	EPR evaluation	3 y	0 (0.0)	0 (0.0)	–
		5MT Fx	EPR evaluation	3 y	1 (0.9)	2 (2.3)	NR
		Radial head/neck Fx	EPR evaluation	3 y	0 (0.0)	0 (0.0)	–
Secondary surgery rate	Ferguson [12]	5MT Fx	EPR evaluation	> 1 y	3 (1.1), 1 non-union, 2 refracture*	2 (0.6), both non-union*	NS
	Mackenzie [9]	5MC Fx	EPR evaluation	3 y	0 (0.0)	0 (0.0)	–
		5MT Fx	EPR evaluation	3 y	1 (0.9, non-union)	2 (2.3), both non-union	NR
		Radial head/neck Fx	EPR evaluation	3 y	0 (0.0)	0 (0.0)	–
Non-comparative							
Non-union rate	Brogan [20]	5MT Fx	EPR and PACS	> 6 m		8 (1.2) Jones type* 5 (0.75) asymptomatic zone 1 fractures in DD cohort	
Secondary surgery rate	Bhattacharyya[18]	Clavicle Fx	EPR evaluation	1y		0 (0.0)	
	Brogan [20]	5MT Fx	EPR and PACS	> 6 m		1 (0.15), symptomatic non-union	
	Jayaram [28]	Radial head/neck Fx	NR	> 6 m		1 (0.5) in DD cohort, malunion Mason II fracture	
Other	Breathnach [19]	Multiple	NR	18–24 m		1 (0.64) poor clinical outcome, referred to physiotherapist	
	Callender [22]	Torus Fx wrist	NR	NR		‘No adverse events or clinically significant complications’	
	Evans [23]	Hand/wrist injuries	Survey	NR		‘No complications of treatment’	
	Ibrahim [27]	Hand injuries	Hand therapist	NR		‘No adverse outcomes’	
	Robinson [31]	Multiple	EPR review	> 1 m		‘No serious adverse outcomes’	

*5MC* fifth metacarpal, *5MT* fifth metatarsal, *d* days, *DD* direct discharge, *EPR* electronic patient record, *Fx* fracture, *m* months, *NR* not reported, *NS* not significant, *Sig.* statistically significant difference, *w* weeks, *y* year(s), * it was not reported if these patients had initially been discharged directly

## Discussion

### Summary of evidence

This systematic review supports the idea that patients with certain simple and stable injuries can be discharged directly from the ED without compromising patient outcome. This suggests that DD offers an opportunity to alleviate fracture clinic workload by reducing unnecessary appointments and consequently healthcare costs. This will allow physicians to spend more time on patients with more complex injuries, teaching, training, or improving standards of care.

Frequently, studies, including several systematic reviews performed recently [32–34], report on the effects of the VFC model as a whole. This model includes both DD of simple stable musculoskeletal injuries, as well as a daily VFC review, consequently including more complex injuries that require follow-up (Fig. 1) [1, 2, 8, 33]. This limits the ability to independently assess the feasibility, efficacy and safety of DD of simple stable musculoskeletal injuries as a solitary concept. To our knowledge, this is the first systematic review to focus exclusively on DD of simple stable musculoskeletal injuries. Herewith, it provides an extensive and critical evaluation of all evidence currently available.

### Logistic outcomes

The DD rate as a proportion of all patients in the ED with a musculoskeletal injury ranged from 18 to 59.5% [1, 2, 8, 14, 30]. This is a remarkably large variation, and despite the studies’ lack of detail on their DD eligibility criteria, this variation is most likely caused by a combination of: (1) differences in the definition of ‘all musculoskeletal injuries’ (i.e., including contusions, wounds, soft-tissue injuries, or not), (2) differences in the kinds of injuries discharged directly, and (3) differences in the period since the DD protocols were first implemented [31]. Regardless of this variation, when implemented, DD will concern a large number of patients with musculoskeletal injuries that are seen frequently in an ED. This is an important factor to determine the logistic level of impact on a fracture clinic. Among the included comparative studies in this systematic review, the mean reduction in the number of follow-up appointments after DD ranged from 1.00 to 1.78 after DD.

### Financial outcomes

All studies that estimated financial effects found DD to reduce healthcare costs. However, cost-analyses were limited to relatively simple estimations of fracture clinic costs, such as material costs, radiology costs and staffing costs. Hence, other important cost categories, such as other healthcare costs, (unpaid) productivity costs, and possibly informal care costs were not included. While it seems evident that healthcare costs reduce when healthcare utilization reduces, these results should be interpreted in a national context, as different healthcare payment systems are in place in each country. Furthermore, full economic evaluations, preferably from a broader healthcare perspective or a societal perspective, are needed to estimate the cost-effectiveness of DD compared with usual practice.

### Patient outcomes

All comparative studies reported ‘as good’ or ‘better’ functional outcome and satisfaction with treatment in the DD cohort compared to patients treated before DD protocols were implemented. The non-comparative studies also reported high levels of satisfaction and satisfactory functional outcome. Of the included studies, Bhattacharyya et al. reported a relatively high mean QuickDASH after DD of patients with a clavicle fracture [18], but this is within the range of the normative values of this questionnaire [35].

Non-union and secondary surgery rate were reported in fifth metatarsal fractures [9, 12, 20], fifth metacarpal fractures [9], radial head fractures [9, 28], and clavicle fractures [18]. These rates were low and comparable in all cohorts. The DD model is established around the idea that a large proportion of patients are well able to manage their recovery independently, if adequately instructed. This model also appreciates that some patients will have concerns or persisting pain, and an even smaller number might develop complications like non-union. However, these problems would have probably also occurred despite routine follow-up, and the majority of patients recover without any issues. Follow-up should, therefore, not solely serve as a safety net to identify those patients with concerns, or complications that might occur in 1–2%. Rather, our results emphasize the importance of instructing patients when to contact the hospital, and of providing an open access helpline in case of any concerns. This helpline should always be part of the DD model, with subsequent face-to-face review in a fracture clinic if necessary.

### Limitations

This review should be regarded in light of the following limitations. First and foremost, there was high clinical and methodological heterogeneity amongst the included studies. As a consequence, we were not able to perform a meta-analysis.

Second, most studies were non-randomized and are therefore prone to selection bias, especially in retrospective cohort studies. Randomization at an individual patient level might not always be feasible for treatment redesigns like DD. However, other methods to reduce confounding effects of systematic differences in baseline characteristics were not used, including institutional cluster-randomization, or advanced statistical techniques such as a propensity score matching or weighting.

Third, only seven studies (27%) assessed functional outcome within the DD cohort using a validated questionnaire, only nine studies (35%) assessed patient satisfaction with treatment and only ten studies (38%) assessed adverse outcomes. Moreover, there was a large variety of other patient-reported outcomes/experiences measured, with methodology of assessment ranging from use of Likert scales, simple yes/no questions, and 1–10 rating scales. Furthermore, with regard to logistic outcomes, the extent to which the included studies assessed whether patients visited their GP or another hospital/clinician for further treatment was limited (Appendix Table S6). However, only non-comparative studies assessed this, while comparative studies would be needed to indicate whether DD increases GP visits or visits to another hospital/clinician.

Fourth, several studies have reported high numbers of patients discharged directly, but this far exceeds the number of patients in which logistic, financial and patient outcomes have actually been evaluated. To illustrate, Glasgow Royal Infirmary have discharged 3802 patients in the first year alone [2], while a later study reported that 30,000 patients were treated successfully since the implementation of their VFC pathway, 65% of which were discharged directly [36]. White et al. studied a cohort of 12,069 patients, 3222 of which were discharged directly [1]. Despite these figures, this systematic review included ‘only’ 2137 patients in the DD cohort to assess logistic outcomes, with even smaller sample sizes for patient outcomes. In other words, there appears to be a gap between clinical practice and evidence base. Additionally, in 62% of the included studies, a clear description of the eligibility criteria for DD was not included and often limited to ‘simple’, ‘minor’ or ‘stable’ injuries. Altogether, this complicates implementation and external validation of DD in other hospitals.

Fifth, most studies were conducted in the UK; hence, the generalizability to other countries might be limited depending on the similarity of their healthcare system with that of the UK, e.g., whether extensive low-threshold public healthcare is available. Different baseline levels of effectiveness of care prior to DD, based on local protocols, might cause logistic outcomes to be different, and patient’ mindset might result in different patient experiences or outcome, as well as patient’s acceptance of DD without further care.

Last, most studies did not report a priori sample size calculations based on the minimal clinical important difference of a predefined primary outcome, such as satisfaction or function. Despite the significant reductions in appointments, sample sizes were often relatively small and, therefore, lacked statistical power to determine a change in patient experience, outcome or complications.

### Future implications

Future studies on DD should be prospective, comparative and include subgroup analysis of each injury eligible for DD. We propose the minimum set of outcome variables of such studies to include: mean number of follow-up appointments, whether patients visited their GP or other hospital, functional outcome using a validated questionnaire, satisfaction using visual analogue scales as well as Likert point scales, and non-union/secondary surgery rates after at least 1 year.

Furthermore, future studies should also focus on fine-tuning the DD treatment protocols by assessment of outcomes within specific patient sub-groups (i.e., based on age, comorbidity, injury subgroup, etc.). If such analyses indicate that specific patient characteristics are predictive of, for example, high levels of return for follow-up, dissatisfaction or low functional outcome, treatment protocols should be adjusted accordingly. Preferably, a multicentre database is be established to this end, as it is likely necessary to have a relatively large sample size in order to have sufficient power to conduct such subgroup analyses.

Based on the high remodelling capacity and low rate of non-union in children [37], it is highly likely that DD is also a safe alternative for several stable paediatric injuries. However, Robinson et al. were the only authors to report on DD of paediatric injuries, other than paediatric clavicle and torus wrist fracture, exclusively within a paediatric cohort [31]. Future studies could focus on the identification of additional minor and stable injuries that can be discharged directly, both in the adult and paediatric population.

## Conclusions

Despite the clinical and methodological heterogeneity of the included studies in this systematic review, DD of several simple and stable injuries seems to be an effective alternative to routine follow-up, which does not seem to compromise patient outcome. Future studies on DD of those as well as other injuries should use a standard set of baseline and outcome variables to improve comparability and facilitate implementation and testing of external validity in other hospitals, especially in countries other than the UK with different healthcare systems.

## Supplementary Information

Below is the link to the electronic supplementary material.

Supplementary file1 (PDF 347 KB)

Supplementary file2 (PDF 36 KB)

## Data Availability

All data are available upon reasonable request.
