# Supplementary material for: Direct discharge of patients with simple stable musculoskeletal injuries as an alternative to routine follow-up: a systematic review of the current literature
Source: Eur J Trauma Emerg Surg. 2021 Sep 16;48(4):2589–605. doi: 10.1007/s00068-021-01784-z (PMC9360121; doi:10.1007/s00068-021-01784-z)
Supplement: Supplementary file 1 — Supplementary file1 (PDF 347 KB) [file 68_2021_1784_MOESM1_ESM.pdf]

Article title: Direct discharge of patients with simple stable injuries as an alternative to routine follow-up: a systematic review of the current literature

Journal title: European Journal of Trauma and Emergency Surgery

Author names: T.H. Geerdink[1*], J. Verbist[1], J.M. van Dongen[2], R. Haverlag[1], R.N. van Veen[1], J.C. Goslings[1]

[1]: Department of Trauma Surgery, OLVG Hospital, Amsterdam, the Netherlands

[2]: Department of Health Sciences, Faculty of Science, Amsterdam Movement Sciences research institute, Vrije Universiteit, Amsterdam, the Netherlands.

*: Corresponding author, Address: Jan Tooropstraat 164, 1061 AE, Amsterdam,

E-mail: t.h.geerdink@olvg.nl; ORCID-ID: 0000-0001-7618-5425

Supplement Statement

The supplementary materials present tables depicting the Patients, Intervention, Control, Outcome strategy (Table S1) used to develop the search strategy (Table S2). Table S3 shows the distribution of the patients within the intervention cohort (i.e. direct discharge straight after the ED attendance (ED DD), after virtual review (VFC DD), or follow-up after virtual review (VFC FU). Immobilization before and after direct discharge protocols were implemented, are summarized in Table S4. Furthermore, the individual scores of the risk of bias assessment are depicted per study in Table S5. Patient-reported outcome and experience measures other than treatment satisfaction and functional outcome using a validated questionnaire are summarized in Table S6.

Appendix tables

Table S1 – Patient, Intervention, Control, Outcome (PICO) strategy used to develop search strategy

PICO	Criteria
Population	Patients of any age presenting to the ED with any type of musculoskeletal injury
Interventions	Direct discharge, either straight from the ED (ED DD) or after virtual review (VFC DD)
Comparison (if applicable)	No direct discharge (i.e. routine follow-up with at least one appointment)
Outcome	Patient direct discharge rate<br>Logistic effects<br>Financial effects<br>Functional outcome<br>Patient reported outcome/experience measures<br>Adverse outcomes

DD, Direct Discharge; ED, Emergency Department; PICO, Patient, Intervention, Control, Outcome; VFC, Virtual Fracture Clinic

Table S2 – Search strategy in MEDLINE

No.	Query	Results
#6	(#1 OR #5)	1,504
#5	(#2 AND #3 AND #4)	958
#4	"Virtual Reality Exposure Therapy"[Mesh] OR "Telemedicine"[Mesh] OR "User-Computer Interface"[Mesh] OR "Remote Consultation"[Mesh] OR electronic referral*[tiab] OR remote consult*[tiab] OR teleconsult*[tiab] OR tele-consult*[tiab] OR virtual*[tiab] OR Re-design*[tiab] OR redesign*[tiab]	174,030
#3	"Patient Discharge"[Mesh] OR "Outpatient Clinics, Hospital"[Mesh] OR "Ambulatory Care Facilities"[Mesh] OR "Ambulatory Care"[Mesh] OR "Outpatients"[Mesh] OR "Appointments and Schedules"[Mesh] OR "Aftercare"[Mesh] OR outpatient*[tiab] OR discharge*[tiab] OR ambulatory[tiab] OR clinic visit*[tiab] OR aftercare*[tiab] OR follow-up[tiab] OR followup[tiab] OR "Referral and Consultation"[Mesh:NoExp] OR visit[tiab] OR visits[tiab] OR appointment*[tiab] OR referral*[tiab]	170,2013
#2	“Fractures, Bone”[Mesh] OR "Wounds and Injuries"[Mesh:NoExp] OR "Arm Injuries"[Mesh] OR "Athletic Injuries"[Mesh] OR "Fractures, Cartilage"[Mesh] OR "Hand Injuries"[Mesh] OR "Finger Injuries"[Mesh] OR "Hip Injuries"[Mesh] OR "Joint Dislocations"[Mesh] OR "Leg Injuries"[Mesh] OR "Multiple Trauma"[Mesh] OR "Shoulder Injuries"[Mesh] OR "Sprains and Strains"[Mesh] OR "Tendon Injuries"[Mesh] OR "injuries" [Subheading] OR fracture*[tiab] OR injur*[tiab] OR orthopedic*[tiab] OR orthopaedic*[tiab]	1,218,483
#1	Fracture clinic*[tiab] OR direct discharge*[tiab] OR ((trauma[tiab] OR fractur*[tiab] OR orthopaedic*[tiab] OR orthopedic*[tiab]) AND (triage clinic*[tiab] OR virtual clinic*[tiab] OR virtual triag*[tiab] OR clinic design*[tiab] OR clinic redesign*[tiab] OR clinic re-design*[tiab] OR service design*[tiab] OR service redesign*[tiab] OR service re-design*[tiab]))	581

Table S3 - Distribution of patients in intervention cohort

Author	Distribution of patients in intervention cohort; n (%)							
	ED DD		ED FU		VFC DD		VFC FU	
	n	%	n	%	n	%	n	%
Abdelmalek [1]	6	22.2	21	77.8				
Bhattacharyya [2]					62	44.9	76	55.1
Breathnach [3]					42	26.8	111	70.7
Brogan [4]					499	75.3	164	24.7
Brooksbank [5]	45	95.7			1	2.15	1	2.15
Evans [6]					54	2.15	228	78.4
Ferguson [7]	194	57.2			86	25.4	59	17.4
Ibrahim [8]					38	38.0	62	62.0
Jayaram [9]	137	67.8			45	22.3	20	9.9
Vardy/Jenkins [10, 11]	2,115	33.1			1,687	26.4	2,583	40.5
Kelly [12]					45	18.2	202	81.8
Little [13]					968	26.1	2,741	73.9
Matthews [14]	18	78.3	5	21.7				
O'Reilly [15]					901	33.3	1,803	66.7
Seewoonarain [16]					33	75.0	11	25.0
White [17]					3,222	26.7	8,847	73.3

Patient distribution in the intervention cohort, indicating whether all patients were discharged directly from the Emergency Department (ED DD), or after Virtual Fracture Clinic review (VFC DD)

Table S4 – Immobilization used before and after implementing direct discharge

Author	Pre-DD	Post-DD
Abdelmalek [1]	Neighbour strapping	Neighbour strapping
Bansal [18]	Plaster slab	Neighbour strapping
Bhattacharyya [2]	Sling	Sling
Breathnach [3]	Unclear	Unclear
Brogan [4]	Non-weightbearing cast	Blackboot
Brooksbank [5]	Unclear	Unclear
Callender [19]	Backslab	Softcast
Evans [6]	Variable	Variable
Ferguson [7]	Variable	Elastic bandage or Velcro boot
Gamble [20]	NR	Neighbour strapping
Gleeson [21] – I	Torus: POP cast; Clavicle: sling	Torus: Velcro wrist splint or soft cast back slab if <2y; Clavicle: sling
Gleeson [22] – II	5MC: ulnar gutter slab; 5MT: below knee back slab plaster cast; Weber A: below knee POP cast	5MC: neighbour strapping; 5MT: tubi grip; Weber A: removable orthosis
Hamilton [23]	Rigid cast	Soft cast
Ibrahim [8]	NR	NR
Jayaram [9]	NR	NR
Jenkins [11]	NR	Removable Velcro splints where required
Kelly [12]	NR	NR
Khan [24]	Rigid cast	Soft cast
Little [13]	NR	NR
Mackenzie [25] – MC5	Variable	Neighbour strapping with/without removable splint
Mackenzie [25] – MT5	Variable	Removable weight-bearing orthosis
Mackenzie [25] – radial head/neck	Variable	Collar and cuff
Matthews [14]	Broad arm sling	Broad arm sling
O'Reilly [15]	NR	Removable splint or cast
Robinson [26]	NR	Finger stall, wrist splint, collar & cuff, walking boot
Seewoonarain [16]	Plaster or Paris or splint	Cast, softcast or splint
Vardy [10]	Unclear	Unclear
White [17]	NR	Removable orthosis

DD, Direct discharge; 5MC, Fifth metacarpal; 5MT, Fifth metatarsal; NR, Not reported; POP, Position-of-protection; y, years;

Table S5 – Risk of bias assessment of the included studies

	Newcastle Ottawa Scale (cohort studies)			Cochrane Collaboration's Risk of Bias Tool (RCTs)							Assessment level
**Author**	**Selection**	**Comparability**	**Outcome**	**Random sequence generation**	**Allocation concealment**	**Blinding of participants/ personnel**	**Blinding of outcome assessment**	**Incomplete outcome data**	**Selective reporting**	**Other sources of bias**	**Risk of bias**
**Comparative**											
Abdelmalek[1]	***		***								Unclear
Bansal [18]	****		***								Low
Ferguson [7]	****		***								Low
Hamilton [23]				Low	Low	High	Low	High	Unclear	Low	High
Kelly [12]	***		**								Unclear
Khan [24]				Unclear	Unclear	High	High	High	Unclear	Unclear	High
Mackenzie [25]	****		***								Low
Matthews [14]	***		***								Unclear
Seewoonarain [16]	****		***								Low
Vardy [10]	****	*	***								Low
**Non-comparative**	**Selection**	**Exposure**	**Outcome**								
Bhattacharyya [2]	**	*	**								Low
Breathnach [3]		*	**								Unclear
Brogan [4]	**	*	***								Low
Brooksbank [5]	**	*	**								Low
Callender [19]	*	*	**								Unclear
Evans [6]	**	*	*								Unclear
Gamble [20]	**	*	**								Low
Gleeson [21] – I		*	*								High
Gleeson [22] – II		*	*								High
Ibrahim [8]	**	*	**								Low
Jayaram [9]	*	*	**								Unclear
Jenkins [11]	**	*	***								Low
Little [13]	**	*	***								Low
O'Reilly [15]	*	*	***								Low
Robinson [26]	**	*	***								Low
White [17]	*	*	***								Low

Risk of bias was assessed using the Newcastle-Ottawa Quality Assessment Scale for cohort studies (Selection, maximum four stars, Comparability, maximum two stars and Outcome, maximum three stars). Using the NOS, an overall score of 7 to 9 stars is considered as low risk of bias, 4 to 6 as unclear risk of bias, and 3 or less as high risk of bias. The NOS was adapted by the authors to be suitable to studies of non-comparative design (Selection, maximum two stars, Exposure, maximum one star, and Outcome, maximum three stars). For the adapted NOS an overall score of 5 to 6 stars is considered as low risk of bias, 3 to 4 as unclear risk of bias, and 2 or less as high risk of bias. Randomized controlled were assessed using Cochrane Collaboration's Risk of Bias Tool. Using this tool, the overall risk of bias is Low if all domains are rated as low, High if at least one domain is assessed as high, and Unclear if at least one domain is assessed as unclear and no domains are assessed as high.

Table S6 - Patient reported outcome- and experience measures

Measure	Author	Assessment: Specification	Reported as	When	How	Control: Score	Resp. rate; (%)	DD: Score	Resp. rate; (%)	Sig.
Satisfaction w/ support / information	Breathnach [3]	Information (in ED), Y/N	Proportion	18-24m	Phone			93% Yes	76.2	
	Bhattacharyya [2]	Information (leaflet), 4P Likert	Proportion	1y	Post			86.4% S	71.0	
	Ferguson [7]	Information (written), 4P Likert*	Proportion	1y	NR	NR		79.6% S or VS	63.7	
	Jayaram [9]	Information (provided), 4P Likert*	Proportion	>6m	Post/phone			95% S	77.0	
	Bansal [18]	Support, scale 1-10	Mean	12w	Phone	5.5 (SD 1.7)	NR	6.4 (SD 2.0)	NR	0.04
Satisfaction w/ treatment	Evans [6]	Process, Likert*	Proportion	NR	Phone			85% VS; 15% S	60.0	
(Satisfaction w/) injury outcome / recovery	Bansal [18]	Lack of terminal extension, Y/N	Proportion	12w	Phone	10% Yes	NR	2.6% Yes	NR	NS
	Bhattacharyya [2]	Satisfaction with outcome, Likert	Proportion	1y	Post			91% VS or S	71.0	
	Breathnach [3]	Satisfaction with outcome, Likert	Proportion	18-24m	Phone			97% A or SA	76.2	
	Ferguson [7]	Satisfaction w/ outcome*	4P Likert	1y	NR	NR		78% S or VS	63.7	
	Gamble [20]	Satisfaction with outcome, Likert	Proportion	>1y	Post/phone			80.6% VS or S	59.0	
	Khan [24]	ROM compared to other side	NR	4-5w	Phone	All full recovery	100 (48)	All full recovery	100	-
	Matthews [14]	Back to normal activities	NR	>6m	NR	NR		All, no residual pain	100	NR
	O'Reilly [15]	Satisfaction with outcome, Likert	Proportion	NR	Phone			97% A or SA	3.6	
Treatment preference	Breathnach [3]	Would prefer face-to-face FU	Y/N	18-24m	Phone			28% Yes	76.2	
	Matthews [14]	Would prefer face-to-face FU	Y/N	>6m	NR	NR		22% Yes	100	NR
	Mackenzie [25] – all	Would prefer face-to-face or DD	Proportion			65% prefer DD	56.4	75% prefer DD	72.3	NR
	Khan [24]	Would prefer treatment again	Y/N	4-5w	Phone	6.3% yes	100	98.6% Yes	100	<0.01
Other clinician	Gamble [20]	Contacted GP	Y/N	>1y	Post/phone			20.4% Yes	59.0	
	Bhattacharyya [2]	Visited GP	Y/N	1y	Post			15.9% Yes	71.0	
	Breathnach [3]	Visited GP	Y/N	18-24m	Phone			6.3% Yes	76.2	
	Gleeson [21] – I	Visited GP	Y/N	3-6w	Phone			0% Yes	45.9	
	Brooksbank [5]	Visited GP / other clinician	Y/N	1y	Post			19% Yes	77.0	
	O'Reilly [15]	Visited GP / other clinician	Y/N	NR	Phone			6% Yes	3.6	
	Jayaram [9]	Visited other clinician*	Y/N	>6m	Post/phone			13% Yes	77.0	
	Ferguson [7]	Visited other hospital*	Y/N	1y	NR	NR		2.8% Yes	63.7	
Helpline use	Breathnach [3]	Aware of availability	Y/N	18-24m	Phone			38% Yes	76.2	
	Jayaram [9]	Aware of availability	Y/N	NR	Phone			94% Yes	3.6	
	Jayaram [9]	Satisfied with, if used, 4P Likert*	Proportion	>6m	Post/phone			93% S	77.0	
	Bhattacharyya [2]	Used	Y/N	1y	Post			9.1% Yes	71.0	
	Ferguson [7]	Used*	Y/N	1y	NR	NR		15.7% Yes	63.7	
	Brooksbank [5]	Used	Y/N	1y	Post			25% Yes	77.0	
	Gamble [20]	Used	Y/N	>1y	Post/phone			3.1% Yes	59.0	
Return-to-work	Bansal [18]		w, mean	12w	Phone	5.0 (SD 2.2)	NR	2.7 (SD 1.5)	NR	<0.01
	Mackenzie [25] – 5MC		w, median	6m	NR	0 (0 to 0)	21.0	0 (0 to 2.0)	53.0	NS
	Mackenzie [25] – 5MT		w, median	6m	NR	2.0 (0 to 4.0)	66.0	1.0 (0 to 2.0)	80.0	NS
	Mackenzie [25] – RH		w, median	6m	NR	0 (0 to 3.0)	80.0	1.0 (0 to 3.5)	80.0	NS
	Mackenzie [25] – RH		w, median	6m	NR	0 (0 to 3.0)	80.0	1.0 (0 to 3.5)	80.0	NS
Return to sport	Mackenzie [25] – 5MC		w, median	6m	NR	2.0 (0 to 4.0)	21.0	2.0 (0 to 4.0)	53.0	NS
	Mackenzie [25] – 5MT		w, median	6m	NR	6.0 (4.0 to 12.0)	66.0	6.0 (4.0 to 8.0)	80.0	NS
	Mackenzie [25] – RH		w, median	6m	NR	6.0 (3.8 to 8.0)	80.0	6.0 (4.0 to 8.0)	80.0	NS

Studies that reported on any type of patient-reported outcome measure excluding functional outcome. 5MC, Fifth metacarpal; 5MT, Fifth metatarsal; DD, Direct Discharge; ED, Emergency Department; FU, follow-up; GP, General Practitioner; m, months; NR, Not reported; NS, Not significant; P, Point; ROM, Range of Movement; RR, Response rate; S, Satisfied; SD, Standard deviation; Sig., Significance level; VS, Very satisfied; w, weeks; y, year(s)

*there was no subgroup analysis exclusively of patients that were discharged directly

References

[1] Abdelmalek A, Harrison E, Scott S. Closed fifth metacarpal neck fractures: Do they need to be seen in fracture clinic? An emergency department pathway for assessment, management and criteria for fracture clinic referral. Trauma. 2014;17:47-51.
[2] Bhattacharyya R, Jayaram PR, Holliday R, Jenkins P, Anthony I, Rymaszewski L. The virtual fracture clinic: Reducing unnecessary review of clavicle fractures. Injury. 2017;48:720-3.
[3] Breathnach O, O'Reilly M, Morrissey K, Conlon B, Sheehan E. Electronic referrals for virtual fracture clinic service using the National Integrated Medical Imaging System (NIMIS). Ir J Med Sci. 2019;188:371-7.
[4] Brogan K, Bellringer S, Akehurst H, Gee C, Ibrahim N, Cassidy L, et al. Virtual fracture clinic management of fifth metatarsal, including Jones', fractures is safe and cost-effective. Injury. 2017;48:966-70.
[5] Brooksbank K, Jenkins PJ, Anthony IC, Gilmour A, Nugent MP, Rymaszewski LA. Functional outcome and satisfaction with a "self-care" protocol for the management of mallet finger injuries: a case-series. J Trauma Manag Outcomes. 2014;8:21.
[6] Evans D, Hardman J, Middleton SD, Anakwe RE. Developing a virtual fracture clinic for hand and wrist injuries. J Hand Surg Eur Vol. 2018;43:893-4.
[7] Ferguson KB, McGlynn J, Jenkins P, Madeley NJ, Kumar CS, Rymaszewski L. Fifth metatarsal fractures - Is routine follow-up necessary? Injury. 2015;46:1664-8.
[8] Ibrahim A, Jordan DJ, Khan MAA, Lowrie AG. The virtual hand clinic: its use in optimizing trauma footfall in a hand trauma unit. 2018. p. 564--6.
[9] Jayaram PR, Bhattacharyya R, Jenkins PJ, Anthony I, Rymaszewski LA. A new "virtual" patient pathway for the management of radial head and neck fractures. J Shoulder Elbow Surg. 2014;23:297-301.
[10] Vardy J, Jenkins PJ, Clark K, Chekroud M, Begbie K, Anthony I, et al. Effect of a redesigned fracture management pathway and 'virtual' fracture clinic on ED performance. BMJ Open. 2014;4:e005282.
[11] Jenkins PJ, Gilmour A, Murray O, Anthony I, Nugent MP, Ireland A, et al. The Glasgow Fracture Pathway: a virtual clinic. Bjj. 2014;395:22--4.
[12] Kelly M, O'Keeffe N, Francis A, Moran C, Gantley K, Doyle F, et al. Connolly Hospital Trauma Assessment Clinic (TAC): a virtual solution to patient flow. Ir J Med Sci. 2020;189:425-9.
[13] Little M, Huntley D, Morris J, Jozsa F, Hardman J, Anakwe RE. The virtual fracture clinic improves quality of care for patients with hand and wrist injuries: an assessment of 3709 patients. J Hand Surg Eur Vol. 2020;45:748-53.
[14] Matthews AH, Boyd M, Bott AR, Metcalfe JE. Improving emergency department management of paediatric clavicle fractures: a complete audit cycle. Br J Hosp Med (Lond). 2014;75:287-9.
[15] M OR, Breathnach O, Conlon B, Kiernan C, Sheehan E. Trauma assessment clinic: Virtually a safe and smarter way of managing trauma care in Ireland. Injury. 2019;50:898-902.
[16] Seewoonarain S, Babu S, Sangoi D, Avasthi A, Ricketts D. Introducing a Virtual Fracture Clinic Increases Efficiency and Reduces Costs in Torus Fracture Management. Pediatr Qual Saf. 2019;4:e202.
[17] White TO, Mackenzie SP, Carter TH, Jefferies JG, Prescott OR, Duckworth AD, et al. The evolution of fracture clinic design : the activity and safety of the Edinburgh Trauma Triage Clinic, with one-year follow-up. Bone Joint J. 2017;99-B:503-7.
[18] Bansal R, Craigen MA. Fifth metacarpal neck fractures: is follow-up required? J Hand Surg Eur Vol. 2007;32:69-73.
[19] Callender O, Koe S. Using Softcast to treat torus fractures in a paediatric emergency department Using Softcast to Treat Torus Fractures in a Paediatric Emergency Department. Irish Medical Journal. 2015:23--5.
[20] Gamble D, Jenkins PJ, Edge MJ, Gilmour A, Anthony IC, Nugent M, et al. Satisfaction and functional outcome with "self-care" for the management of fifth metacarpal fractures. Hand (N Y). 2015;10:607-12.
[21] Gleeson L, Murray O. Fracture Clinic Redesign in the Ed: Breaking the Mould. Emergency Nurse New Zealand. 2016:P14--P7.
[22] Gleeson L, Murray O. Fracture Clinic Redesign in the Ed: Breaking the Mould. Part Two: Weber A, 5TH Metacarpal and 5TH ETATARSAL FRACTURES. Emergency Nurse New Zealand. 2016:P12--P5.
[23] Hamilton TW, Hutchings L, Alsousou J, Tutton E, Hodson E, Smith CH, et al. The treatment of stable paediatric forearm fractures using a cast that may be removed at home: comparison with traditional management in a randomised controlled trial. Bone Joint J. 2013;95-B:1714-20.
[24] Khan KS, Grufferty A, Gallagher O, Moore DP, Fogarty E, Dowling F. A randomized trial of 'soft cast' for distal radius buckle fractures in children. Acta Orthopaedica Belgica. 2007;73:594--7.
[25] Mackenzie SP, Carter TH, Jefferies JG, Wilby JBJ, Hall P, Duckworth AD, et al. Discharged but not dissatisfied: outcomes and satisfaction of patients discharged from the Edinburgh Trauma Triage Clinic. Bone Joint J. 2018;100-B:959-65.
[26] Robinson PM, Sim F, Latimer M, Mitchell PD. Paediatric fracture clinic re-design: Incorporating a virtual fracture clinic. Injury. 2017;48:2101-5.
