# Supplementary material for: Direct discharge of patients with simple stable musculoskeletal injuries as an alternative to routine follow-up: a systematic review of the current literature
Source: Eur J Trauma Emerg Surg. 2021 Sep 16;48(4):2589–605. doi: 10.1007/s00068-021-01784-z (PMC9360121; doi:10.1007/s00068-021-01784-z)
Supplement: Supplementary file 2 — Supplementary file2 (PDF 36 KB) [file 68_2021_1784_MOESM2_ESM.pdf]

Article title: Direct discharge of patients with simple stable injuries as an alternative to routine follow-up: a systematic review of the current literature

Journal title: European Journal of Trauma and Emergency Surgery

Author names: T.H. Geerdink[1*], J. Verbist[1], J.M. van Dongen[2], R. Haverlag[1], R.N. van Veen[1], J.C. Goslings[1]

[1]: Department of Trauma Surgery, OLVG Hospital, Amsterdam, the Netherlands

[2]: Department of Health Sciences, Faculty of Science, Amsterdam Movement Sciences research institute, Vrije Universiteit, Amsterdam, the Netherlands.

*: Corresponding author, Address: Jan Tooropstraat 164, 1061 AE, Amsterdam,

E-mail: t.h.geerdink@olvg.nl; ORCID-ID: 0000-0001-7618-5425

Supplement Statement

The following table includes the PRISMA checklist for reporting systematic review.

Section/topic	#	Checklist item	Reported on page #
**TITLE**			1
Title	1	Identify the report as a systematic review, meta-analysis, or both.	1
**ABSTRACT**			2
Structured summary	2	Provide a structured summary including, as applicable: background; objectives; data sources; study eligibility criteria, participants, and interventions; study appraisal and synthesis methods; results; limitations; conclusions and implications of key findings; systematic review registration number.	2
**INTRODUCTION**			3
Rationale	3	Describe the rationale for the review in the context of what is already known.	3
Objectives	4	Provide an explicit statement of questions being addressed with reference to participants, interventions, comparisons, outcomes, and study design (PICOS).	3
**METHODS**			4-6
Protocol and registration	5	Indicate if a review protocol exists, if and where it can be accessed (e.g., Web address), and, if available, provide registration information including registration number.	4
Eligibility criteria	6	Specify study characteristics (e.g., PICOS, length of follow-up) and report characteristics (e.g., years considered, language, publication status) used as criteria for eligibility, giving rationale.	4
Information sources	7	Describe all information sources (e.g., databases with dates of coverage, contact with study authors to identify additional studies) in the search and date last searched.	4
Search	8	Present full electronic search strategy for at least one database, including any limits used, such that it could be repeated.	4-5
Study selection	9	State the process for selecting studies (i.e., screening, eligibility, included in systematic review, and, if applicable, included in the meta-analysis).	5
Data collection process	10	Describe method of data extraction from reports (e.g., piloted forms, independently, in duplicate) and any processes for obtaining and confirming data from investigators.	5
Data items	11	List and define all variables for which data were sought (e.g., PICOS, funding sources) and any assumptions and simplifications made.	5**Error! Bookmark not defined.**
Risk of bias in individual studies	12	Describe methods used for assessing risk of bias of individual studies (including specification of whether this was done at the study or outcome level), and how this information is to be used in any data synthesis.	5
Summary measures	13	State the principal summary measures (e.g., risk ratio, difference in means).	5-6

Synthesis of results	14	Describe the methods of handling data and combining results of studies, if done, including measures of consistency (e.g., I^2) for each meta-analysis.	6

Section/topic	#	Checklist item	Reported on page #
Risk of bias across studies	n/a	Specify any assessment of risk of bias that may affect the cumulative evidence (e.g., publication bias, selective reporting within studies).	n/a
Additional analyses	n/a	Describe methods of additional analyses (e.g., sensitivity or subgroup analyses, meta-regression), if done, indicating which were pre-specified.	n/a
**RESULTS**			7-8
Study selection	15	Give numbers of studies screened, assessed for eligibility, and included in the review, with reasons for exclusions at each stage, ideally with a flow diagram.	7
Study characteristics	16	For each study, present characteristics for which data were extracted (e.g., study size, PICOS, follow-up period) and provide the citations.	7
Risk of bias within studies	17	Present data on risk of bias of each study and, if available, any outcome level assessment (see item 12).	7
Results of individual studies	18	For all outcomes considered (benefits or harms), present, for each study: (a) simple summary data for each intervention group (b) effect estimates and confidence intervals, ideally with a forest plot.	7-8
Synthesis of results	n/a	Present results of each meta-analysis done, including confidence intervals and measures of consistency.	n/a
Risk of bias across studies	n/a	Present results of any assessment of risk of bias across studies (see Item 15).	n/a
Additional analysis	n/a	Give results of additional analyses, if done (e.g., sensitivity or subgroup analyses, meta-regression [see Item 16]).	n/a
**DISCUSSION**			9-11
Summary of evidence	19	Summarize the main findings including the strength of evidence for each main outcome; consider their relevance to key groups (e.g., healthcare providers, users, and policy makers).	9
Limitations	20	Discuss limitations at study and outcome level (e.g., risk of bias), and at review-level (e.g., incomplete retrieval of identified research, reporting bias).	10
Conclusions	21	Provide a general interpretation of the results in the context of other evidence, and implications for future research.	11
**FUNDING**			1

Funding	22	Describe sources of funding for the systematic review and other support (e.g., supply of data); role of funders for the systematic review.	1

From: Moher D, Liberati A, Tetzlaff J, Altman DG, The PRISMA Group (2009). Preferred Reporting Items for Systematic Reviews and Meta-Analyses: The PRISMA Statement. PLoS Med 6(7): e1000097. doi:10.1371/journal.pmed1000097
